# Attribute-Based Proxy Re-Encryption with Keyword Search

**DOI:** 10.1371/journal.pone.0116325

**Published:** 2014-12-30

**Authors:** Yanfeng Shi, Jiqiang Liu, Zhen Han, Qingji Zheng, Rui Zhang, Shuo Qiu

**Affiliations:** 1 School of Computer and Information Technology, Beijing Jiaotong University, Beijing, China; 2 The State Key Laboratory of Information Security, Institute of Information Engineering, Chinese Academy of Sciences, Beijing, China; 3 Department of Computer Science, University of Texas at San Antonio, San Antonio, Texas, United States of America; Tianjin University of Technology, China

## Abstract

Keyword search on encrypted data allows one to issue the search token and conduct search operations on encrypted data while still preserving keyword privacy. In the present paper, we consider the keyword search problem further and introduce a novel notion called *attribute-based proxy re-encryption with keyword search* (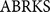), which introduces a promising feature: In addition to supporting keyword search on encrypted data, it enables data owners to delegate the keyword search capability to some other data users complying with the specific access control policy. To be specific, 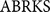 allows (i) the data owner to outsource his encrypted data to the cloud and then ask the cloud to conduct keyword search on outsourced encrypted data with the given search token, and (ii) the data owner to delegate other data users keyword search capability in the fine-grained access control manner through allowing the cloud to re-encrypted stored encrypted data with a re-encrypted data (embedding with some form of access control policy). We formalize the syntax and security definitions for 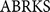, and propose two concrete constructions for 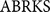: key-policy 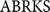 and ciphertext-policy 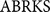. In the nutshell, our constructions can be treated as the integration of technologies in the fields of attribute-based cryptography and proxy re-encryption cryptography.

## Introduction

Cloud computing platforms assemble vast computational resources and make them available to users as a service. The cloud users can outsource their heavy computation tasks and/or storage to cloud providers while still enjoying promising properties, e.g., low maintenance cost and pervasive accessing. While it is promising, cloud computing also confronts many challenges against data privacy/system vulnerabilities [1]–[3] and service quality [4], [5]. One possible solution to prevent these problems is to use the private cloud, where the underlying infrastructure (i.e., servers, network and storage) is owned and operated by the cloud users themselves. However, this might depress the benefits bringing from the cloud computing, when comparing with the public cloud that is more reliable, elastic (i.e., computational resources can be increased and decreased quickly) and cost-saving. As such, individual and organizations are considering migrating from their owned infrastructure to the public cloud.

In order to preserve data privacy against any possible attacks in the public cloud, it is inevitable for data owners to encrypt their data before outsourcing it to the cloud, which might hinder the data usage. For example, how the data owner can search on their outsourced encrypted data? How the data owner can delegate his search capability to other users in a fine-grained manner? In this paper, we continue the line of keyword search on encrypted data and attempt to solve the above questions simultaneously.

To explain the motivation for solving the above questions, we consider the following motivational application: The data owner, say Alice, encrypted her personal health data that was collected by sensors attached her and outsourced the encrypted data to the cloud. In order to facilitate the examination on health condition, Alice may need to share the encrypted data with professionals, e.g. doctors that work in some specific department, so that the professionals can retrieve qualified records from the cloud. In order to assure that only certain professionals satisfying some policy can conduct keyword search and retrieve corresponding encrypted data of their interests, Alice needs to delegate keyword search capability by specifying the fine-grained access control policy.

A straightforward solution toward the above questions can work as follows: the data owner encrypts his data with attribute-based encryption, and issues proper keys to data users so that only authorized data users can access these encrypted data. Unfortunately, solutions based on attribute-based encryption in the literature do not support keyword search. That is, even satisfying the access control policy, the authorized user has to download entire encrypted data, rather than portion of encrypted data of his interest, which will bring in huge communication overhead. In light of this, we propose a novel notion, dubbed attributed-based proxy re-encryption with keyword search (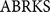), allowing data owners to grant keyword search capability to authorized users complying with access control policies.

### Our Contribution

We introduce a novel notion called attribute-based proxy re-encryption with keyword search (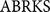), which allows a data owner to delegate keyword search capability over his encrypted data to authorized users by while complying with access control policies. We formally define its syntax and rigorously formalize the security definitions. We present two flavors of 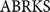 constructions, key-policy 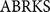 and ciphertext-policy 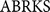, the security of which are based on the standard Multilinear Decisional Diffie-Hellman Assumption in the random oracle model. Our solutions perfectly solve the motivation example and enjoy three distinctive properties: (i) The data owner could conduct keyword search on outsourced encrypted data; (ii) The data owner could delegate keyword search capability to users by specifying fine-grained access control policies so that only authorized users satisfying the access control policy can conduct keyword search; and (iii) There is no interaction happening between data owners and users. Moreover, the tedious work, e.g., performing keyword search and re-encrypting encrypted data, can be outsourced to the cloud without compromising data privacy.

### Related Work

Here we briefly survey the works that are relevant to the problem we attempt to solve in this paper, while cannot solve it. We summarize the features of the most relevant techniques, proxy re-encryption with keyword search, attribute-based encryption, attribute-based encryption with keyword search and attribute-based proxy re-encryption, and compare them with our 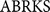 solutions as shown in Table 1.

**Table 1:** Property summary for PRES, ABE, ABPRE, ABKS in the literature and the solution in this paper.

Scheme	Proxy Re-encryption	Keyword Search	Access Control
PRES [6]–[11]	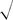	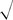	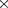
ABE [12]–[18]	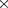	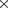	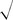
ABKS [19], [20]	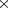	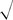	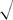
ABPRE [21]–[26]	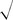	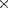	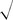
ABRKS(Our solution)	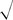	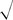	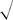

#### Proxy Re-encryption with Keyword Search

Proxy re-encryption with keyword search (PRES) was introduced in [6], which allows a data owner to delegate keyword search capability to other users. PRES was further revised by [7] and/or enhanced by various papers, e.g., [8]–[11]. However, all these PRES solutions only considered coarse-grained access control enforcement, i.e., delegating the search capability to one specific authorized user. In contrast, we consider the fine-grained access control enforcement when the data owner needs to delegate search capability in this paper.

#### Attribute-based Encryption

Attribute-based encryption (ABE) was first introduced by [12], which is to specify fine-grained access control on encrypted data, such that only data users with proper credentials (i.e., satisfying the access control policy) can decrypt the ciphertexts. There are two flavors of ABE depending on the manner of associating access control policy: key-policy ABE (KP-ABE) [13]–[15] associates the decryption key with the access control policy and ciphertext-policy ABE (CP-ABE) associates the ciphertext with the access control policy [16]–[18]. While ABE allows data owners to achieve fine-grained access control enforcement on encrypted data, unfortunately it cannot support keyword search.

#### Attribute-based Encryption with Keyword Search

The concept of attribute-based encryption with keyword search (ABKS) was introduced by [19] and [20] independently. It allows data owner to grant search capability to authorized users by specifying fine-grained access control when encrypting plaintext. However, it does not support the data owner delegating search capability to authorized users when encrypted data were stored in the cloud.

#### Attribute-based Proxy Re-encryption

Attribute-based proxy re-encryption (ABPRE) was introduced by [21] and enriched by [22]–[26] with various features. However, these solutions do not support the function of keyword search on encrypted data. Generally speaking, the solution in this paper can be regarded as an extension to ABPRE with the feature of keyword search on encrypted data.

## Preliminary

### Cryptographic Assumptions

#### Multilinear Maps

The concept of multilinear maps was introduced in [27] and came to reality thanks to [28], [29]. Given a security parameter 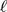 and an 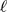-bit prime 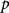, a 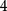-multilinear map consists of 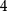 cyclic groups (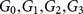) of order 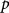, and 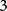 mappings 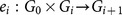, 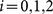. The 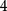-multilinear map should satisfy the following properties with respect to 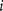, 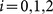: (i) Given that 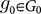 is a generator of 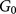, then 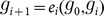 is a generator of 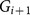; (ii) 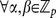, 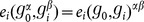; and (iii) 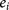 can be efficiently computed.

#### 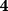-Multilinear Decisional Diffie-Hellman Assumption (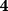-MDDH)

Given the 4-multilinear map and 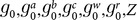, where 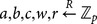 that are unknown, 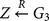, 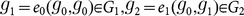 and 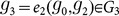, there exists no probabilistic polynomial algorithm 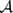 that can determine whether 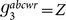 or not with a non-negligible advantage with respect to security parameter 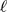, where the advantage is defined as 
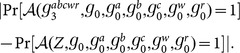



### Access Control Policy

#### Linear Secret Sharing Scheme

A linear secret sharing scheme (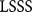) can be used to represent an access control policy 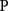 via 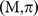, where 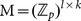 is an 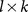 dimensional matrix with entries belonging to 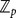 and 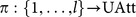 is an injective function that maps a row into an attribute. Given an attribute set 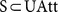 where 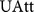 is the attribute universe, we denote 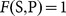 if 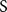 satisfies the access control policy 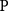. Specifically, an 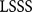 consists of two algorithms:


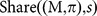
: This algorithm is to distribute a secret value 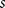 with respect to the attributes specified by 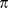: It selects 
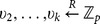
, sets 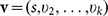 and computes 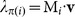 where 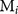 is the 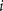
th row of 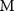. Then it assigns secret share 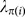 to the attribute 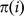.


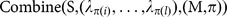
: This algorithm is to assemble the secret value from the secret shares associated with respect to the attributes: It selects a subset 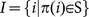 such that the attribute set 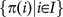 satisfies the access control policy 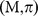, and then computes the coefficients 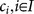 such that 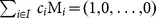. The recovered secret will be 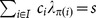.

The correctness of algorithm 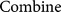 is guaranteed by the following lemma:

**Lemma 1** ([17]) *Let* 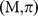 *be an* 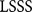 *representing an access control policy* 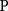 *. For all attributes in* 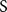 *that do not satisfy* 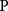 *, there is a polynomial-time algorithm that outputs vector* 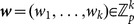 *such that* 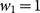 *and* 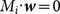 *for all* 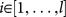 *, where* 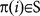 *.*

## Definition

### System Model

**Figure 1:**
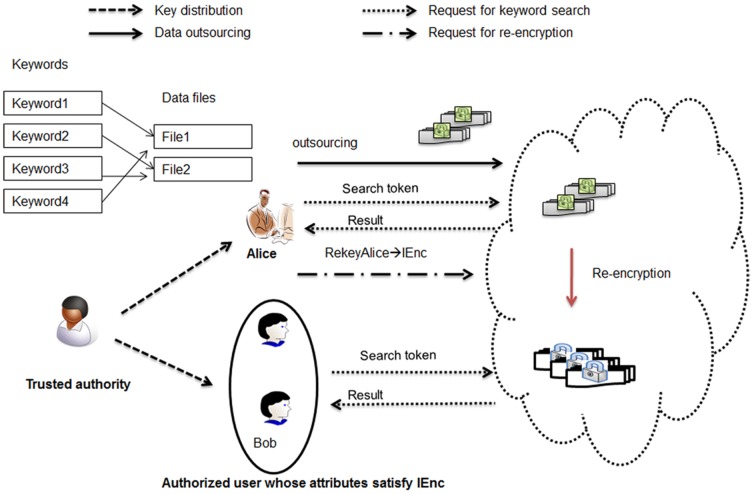
System model of attribute-based access control for proxy re-encryption with keyword search.

The system model of attribute-based proxy re-encryption with keyword search is shown in Fig. 1, consisting of three parties: the trusted authority, the cloud server and cloud users that can be either data owner or data users wishing to share the data owner's data. The trusted authority is responsible for initiating system public parameters and issuing private keys to cloud users with respect to their attributes. A data owner (say Alice) encrypts her data and the keyword index and outsource the encrypted data and the associated encrypted keyword index to the cloud server. Moreover, the data owner can retrieve encrypted data of her interest by issuing a search token with respect to some keyword to the cloud. On the other hand, the data owner is capable of granting search capability to other authorized users by issuing re-encryption keys (which is associated with access control policies) to the cloud. The cloud server provides storage and computation service for cloud users. Especially, the cloud server can transform the stored encrypted data with re-encryption keys from the data owner, so that the authorized data user (say Bob) is able to generate search tokens and ask the cloud server to conduct keyword search on the re-encrypted data for retrieving encrypted data of his interest. In this model, we assume that the data owner and data users require no direct interaction.

### Functional Definition

We now present the formal definition of attribute-based proxy re-encryption with keyword search, which consists of two variants: key-policy 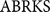 (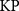 - 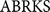) whose private keys are associated with access control policies, and ciphertext-policy 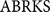 (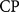 - 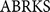) whose ciphertexts after re-encryption are associated with access control policies. To unify the presentation, let 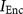 denote the input of the encryption function 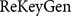 and 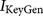 denote the input of the key generation function 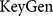. Therefore, 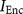 and 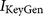 respectively correspond to an attribute set and an access policy in 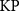 - 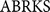, whereas 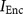 and 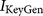 respectively correspond to an access policy and an attribute set in 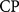 - 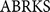. We denote 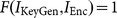 if and only if 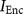 satisfies 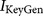 in 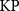-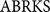 or 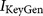 satisfies 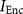 in 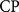-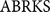.

To be specific, an 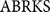 scheme consists of algorithms as follows:


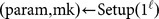
: Taking as input a security parameter 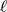, this algorithm is run by the trusted authority to initiate the public parameter 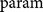 and a master private key 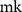.


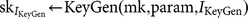
: Taking as input 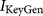, the master key 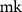 and public parameter 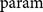, this algorithm is run by the trusted authority to issue a private key 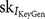 associated with 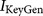 for a data user.

(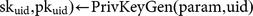: Taking as input a user's identity 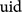, the master key 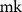 and public parameter 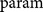, this algorithm is run by the trusted authority to generate a pair of keys (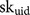,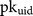).


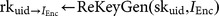
: Taking as input a user's private key 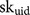 and 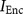, this algorithm is run by the data owner to generate the re-encryption key 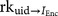.


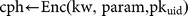
: Given a keyword 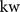, the public parameter 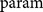, and the data owner's public key 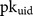, this algorithm is run by the data owner to output an original ciphertext 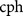.


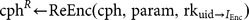
: Given a ciphertext of 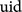, the public parameter 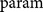, and a re-encryption key 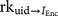, this algorithm is run by the cloud server to output a re-encrypted ciphertext 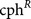.


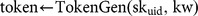
: This algorithm is run by the data owner to generate a token 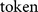, which can be used to conduct the search operation over original encrypted keywords.


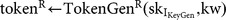
: This algorithm is run by a data user to generate a token 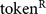, which can be used to conduct the keyword operation over re-encrypted keywords.


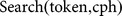
: This algorithm, run by the cloud server, returns 1 if the original encrypted keyword 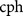 and the token 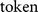 correspond to the same keyword; otherwise it returns 0.


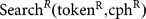
: This algorithm, run by the cloud server, returns 1 if (i) 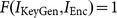 and (ii) the re-encrypted keyword 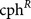 and the token 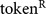 correspond to the same keyword; otherwise it returns 0.

**Correctness** We say an 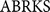 scheme is secure if, for 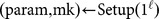, (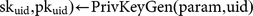, 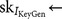 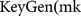, 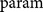, 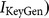, then the follows should hold:

- Given 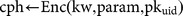 and 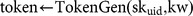, 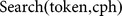 always returns 1;
- Given 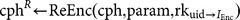 and 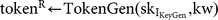, where 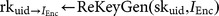, 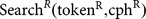 always returns 1 if 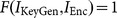.

### Security Definitions

The security of 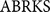 requires that the ciphertexts and tokens leak nothing about the underlying keywords. Informally, the adversary is allowed to query ciphertext of any plaintext and tokens except those corresponding to two keywords in the challenge phase. We expect that the adversary cannot distinguish the challenge ciphertext that is generated from one of keywords 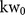 and 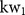. To formalize aforementioned security notion, we define the selective chosen keyword security game as follows. Note that in our corruption model, the adversary is not allowed to get the re-encryption key from uncorrupted users to corrupted users. Note that in our security model we consider the static corrupted model in the sense that the set of corrupted users has to be selected in the setup phase.

### Setup

The adversary 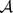 selects a set of corrupted users denoted by 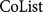 and 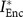, and sends them to the challenger. The challenger runs 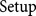 to produce 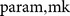, sends 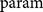 to 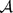 and keeps 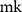 private.

### Phase 1


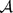
 can query the following oracles in polynomially many times:

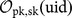
: It runs 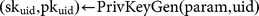. If 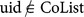, it returns the public key 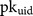 to 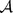; otherwise 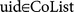, then it returns the key pair 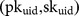 to 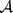. We assume that before querying oracles 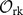, 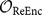 and 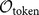, the user's private key 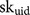 has been generated.

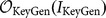
: If 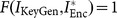, it aborts. Otherwise, it runs 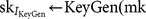, 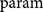, 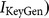 and returns the private key 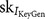 to 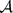.

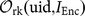
: If 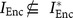 and 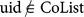, it aborts because it is not allowed to query re-encrypted key from an uncorrupted user to 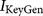 where 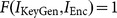. Otherwise, it runs 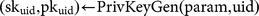 and 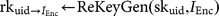, and returns the re-encryption key 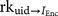.

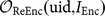
: It runs 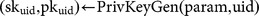, 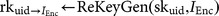 and 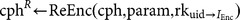, and returns re-encrypted keyword 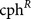 to 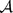.

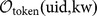
: It runs 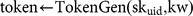, and returns the token 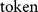 for 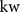 over original encrypted keyword to 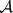.

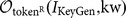
: It runs 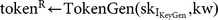 and returns the token 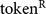 for 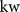 over re-encrypted keyword to 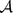.

### Challenge


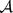
 selects an uncorrupted user 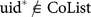 and two equal-length keywords (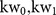), where (i) 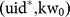 or 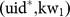 have never been queried on 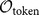 and (ii) if 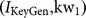, then 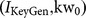 and 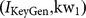 have not been queried to 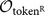. 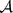 sends them to the challenger. The challenger selects 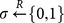, runs 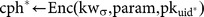 and forwards 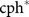 to 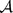.

### Phase 2


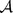
 queries the oracles the same as Phase 1 except that

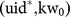
 and 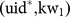 are not allowed to query on 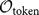.
- If 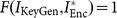, then 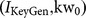 and 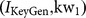 should not been queried to 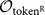

### Guess


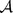
 outputs a guess 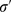. We say that 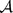 wins the game if 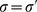.

### Definition 1

*We say that an* 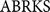 *scheme achieves selective security against chosen-keyword attack if any probabilistic polynomial-time adversary* 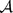 *wins the selective security game defined above with a negligible advantage with respect to the security parameter* 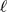 *, where the advantage is defined as* 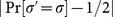 *.*

## Methods

### The Basic Idea

**Figure 2:**
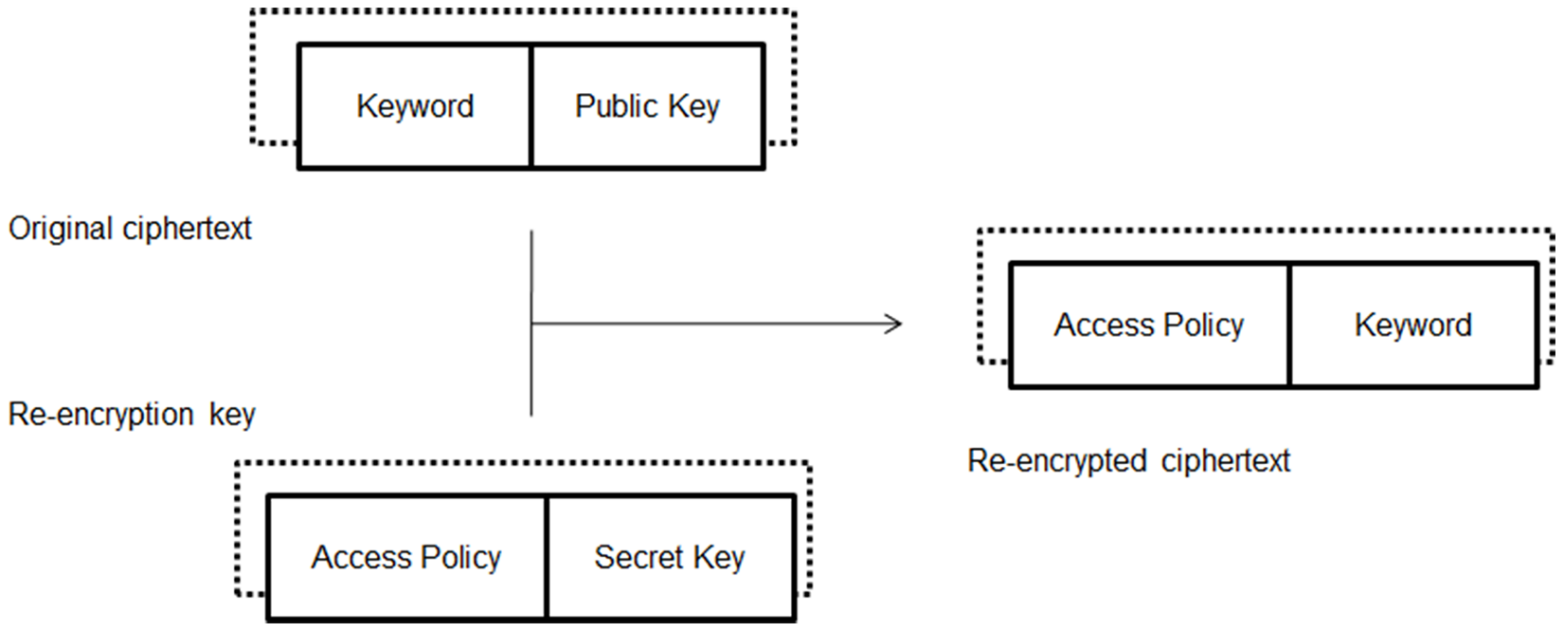
The high level idea of enabling keyword search over re-encrypted ciphertext by re-encryption.

In our 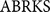 scheme, the critical part is how to support keyword search over re-encrypted ciphertexts while being able to enforce access control. In order to achieve this, our intuition (shown in Fig. 2) is to compose the re-encrypted ciphertext with two components: one is associated with the keyword and is transformed from original encrypted ciphertext; the other one is associated with the access control policy and can be derived from the re-encryption key where the access control policy is determined by the data owner.

### 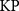 - 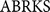 Construction

Recall that an access control policy is represented by 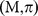, where 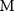 is an 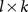 dimensional matrix and 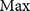 is the maximum number of attributes associated with a ciphertext. Note that let 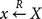 denote selecting element 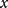 from the set 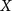 uniformly at random. The 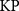-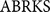 scheme can be constructed as follows:


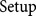
(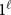): Given the security parameter 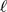, the algorithm generates the public parameters and the master key as follows:

- Generate a 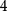 multi-linear map: 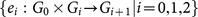, where (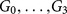) are cyclic groups of order 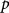 respectively. Let 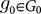 be a generator of 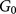, and 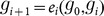 be the generator of 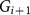 for 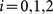.
- Let 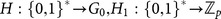 be two secure hash functions modeled as random oracles.
- Let 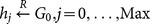 and define a function 
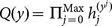
 where 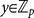.
- Choose 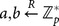 and set the public parameters and master key as

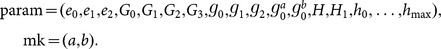


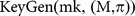
: Given an access control policy 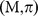, this algorithm generates the private key as follows:

- Select 
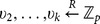
, set 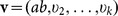, and compute 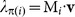 for 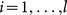.
- For each 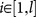, select 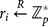 and set

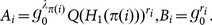

- The private key is set to

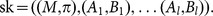


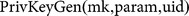
: Given a user's identity 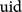, this algorithm selects 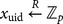 and sets 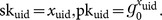


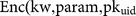
): Given a keyword 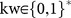, this algorithm selects 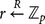, and sets 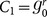 and 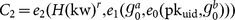. It sets the original encrypted keyword as 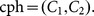


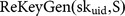
: Taking as input the data owner's private key 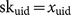 and an attribute set 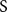, this algorithm generates the re-encryption key as follows:

- Select 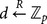 and set 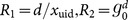.
- Set 
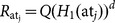
 for each 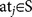.
- Set the re-encryption key as 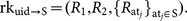

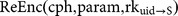
: Given the original ciphertext 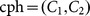 and the re-encryption key 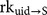, it computes 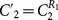 and re-encrypts 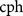 to 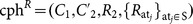.


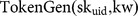
: Given the private key 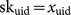 of data user 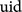 and a keyword 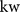, this algorithm sets the token for the keyword 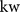 over original encrypted keywords as 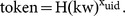


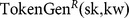
: Given the data user's private key 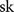, this algorithm computes 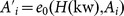 and 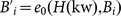 for 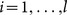. It sets the token for the keyword 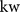 over re-encrypted keywords as 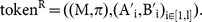


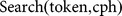
: Given the original encrypted keyword 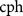 and a token 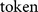 generated by the data owner, this algorithm outputs 1 if 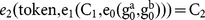, and 0 otherwise.


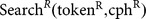
: Given the re-encrypted keyword 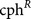 and a token 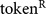 generated by the data users, the search can be done as follows:

- If the attribute set 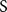 associated with 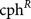 satisfies the access control policy specified by 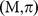 associated with 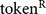, compute 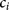 such that 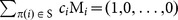, and let
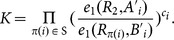
If 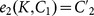, output 1 and 0 otherwise.

- Otherwise, output 0.

### Correctness

The correctness of the 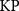 - 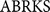 scheme can be verified as follows:

If 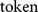 and the original ciphertext correspond to the same keyword, we have 
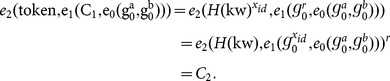
If the attribute set 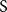 satisfies the access control policy specified by 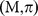, and 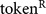 and the re-encrypted ciphertext correspond to the same keyword, 
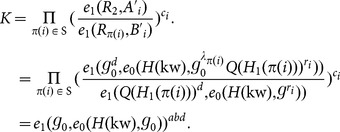
such that 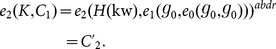



### 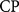 - 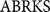 Construction

We also elaborate the construction of the 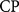-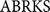 scheme as follows.


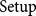
 (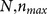): This algorithm takes as input 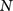, the number of attributes in the system and 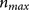 the maximum of columns of 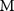. It generates the public parameters and the master key as follows:

- Generate a 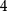 multi-linear map: 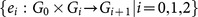, where (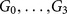) are cyclic groups of order 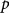 respectively. Let 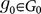 be a generator of 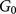, and 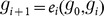 be a generator of 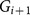 for 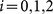.
- Select elements 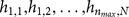 from 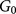 uniformly at random.
- Let 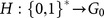 be a secure hash function modeled as a random oracle.
- Select 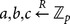 and set the public parameters and master key as

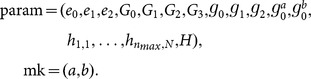


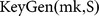
: Given an attribute set 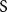, this algorithm generates the private key as follows:

- Choose 
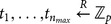
, and set 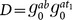.
- For each 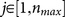 set 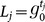 and for each 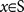 set 
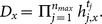

- The private key is set to

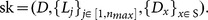


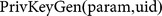
: This algorithm is the same as 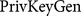 algorithm in 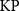 - 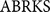.


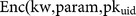
): This algorithm is the same as 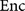 algorithm in 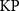 - 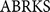.


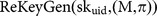
: Taking as input a user's private key 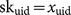 and an access control policy 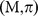, where 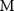 is an 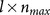 matrix (If the number of columns of 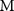 is 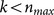, it can simply “pad out” the rightmost 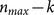 columns with zeros.), this algorithm generates the re-encryption key as follows:

- Select 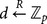 and set 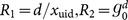.
- Choose 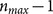 random elements 
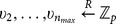
, let the vector 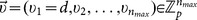, and set 
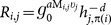
 for each 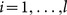 and 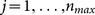.
- Set the re-encryption key as 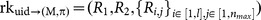.

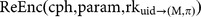
: Given an original encrypted keyword 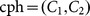 and the re-encryption key 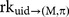, the algorithm computes 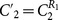 and re-encrypts 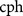 to 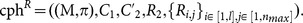


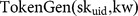
: This algorithm performs as same as 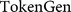 algorithm in 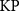 - 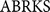.


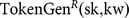
: Given credentials 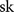, this algorithm computes 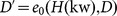, 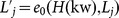 for 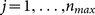 and 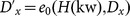 for 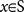. It sets the token for the keyword 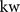 over re-encrypted keywords as 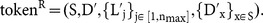


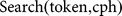
: This algorithm performs the same as 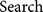 algorithm in 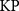 - 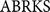.


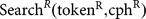
: Given the re-encrypted keyword 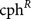 and a token 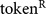 generated by the data users, the search can be done as follows:

- If the attribute set 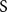 associated with 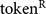 satisfies the access control policy specified by 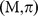 associated with 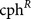, compute 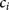 such that 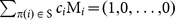, and let
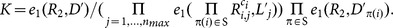
If 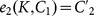, output 1 and 0 otherwise.

- Otherwise, output 0.

### Correctness

The correctness of the 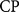-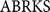 scheme can be verified similar to that of 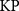-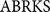 scheme.

## Discussion

### 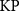 - 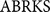 Security

#### Theorem 1

*Assume that* 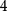 *-MDDH assumption holds, our* 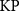 *-* 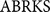 *scheme achieves selective security against chosen-keyword attack in the random oracle model.*

*Proof*: The proof strategy is to reduce the security of our construction to the hardness of 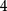-MDDH assumption. That is, we show that if there exists a probabilistic polynomial time adversary 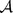 breaking selective security game of 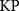-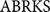 against chosen-keyword attack with a non-negligible advantage 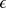, then we can simulate a challenger solving 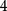-MDDH problem with a non-negligible advantage 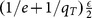, where 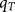 is a polynomial large number, which should be larger than the number of oracle queries for 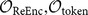 and 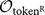.

Given an instance of 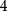-MDDH problem 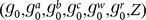, where 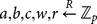 are unknown, the challenger simulates the game as follows:

#### Setup


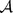
 selects a set of corrupted users denoted by 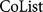 and an attribute set 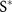, and sends them to the challenger. The challenger generates the public parameters and master key as follows:

- Given the attribute set 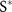, let 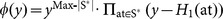, which can be rewritten as 
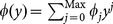
, where 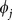 is the coefficient of 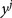 and therefore 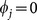 for 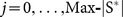.
- Select 
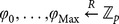
, and define 
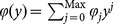
.
- Let 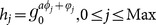, and define 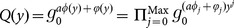.
- The public parameters is set to
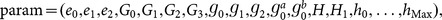
by implicitly setting the master private key 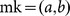.

Moreover, the challenger simulates the oracles 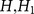 as follows:

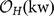
: Given a keyword 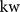, it proceeds as follows:
  1. If 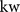 has not been queried before, then select 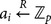 and toss a random coin 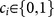 with the probability that 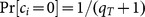, where 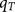 is a polynomial large number. We require that 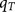 should be larger than the number of oracle queries for 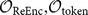 and 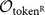. If 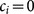, then compute 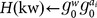; Otherwise, compute 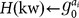. Add 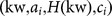 to 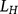 and return 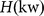.
  2. Otherwise, retrieve 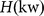 from 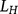 with respect to 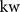 and return 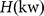.

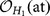
: If the attribute 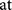 has not been queried before, select 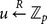, set 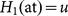, and add 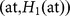 to the list 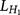. Otherwise, retrieve 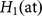 from 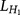 with respect to 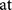. Eventually, it returns 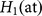.

#### Phase 1


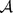
 can query the following oracles in polynomially many times:

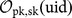
: Given a user identity 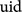, the challenger proceeds as follows:
  1. If 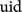 has been queried before, retrieve (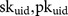) from 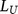 with respect to 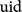 and return (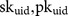).
  2. Otherwise, select 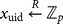. If 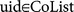, compute 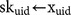 and 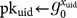; otherwise set 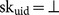 and 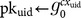. Finally add 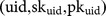 to 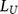 and return (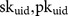).

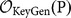
: Given an access control policy 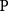 specified by 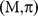, the challenger proceeds as follows:
  1. If 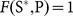, then abort.
  2. Otherwise, because 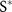 does not satisfy the access structure 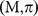, there exists a vector 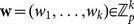 such that 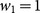 and 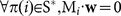. Choose 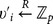 for 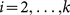, and set 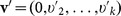. By implicitly setting 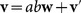, it generates 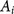 and 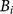 as follows:
    1. If 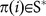, select 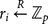 compute 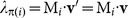, and set 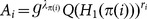 and 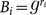.
    2. Otherwise, select 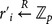 and compute

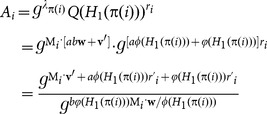


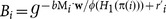
by implicitly setting 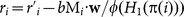.

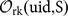
: Given a user identity 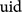 and an attribute set 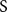, the challenger proceeds as follows:
  1. If 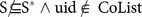, then abort.
  2. If 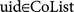, choose a random 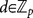 and set

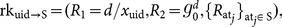
where 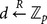 and 
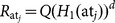
.

  - Otherwise, choose 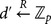 and set
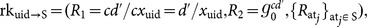
by implicitly letting 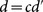. Note that 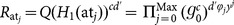, since 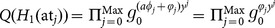.

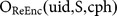
: Given a user identity 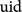, an original encrypted keyword 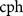 and an attribute set 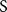, the challenger proceeds as follows:
  1. If 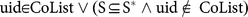, it queries 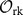 with (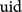, 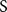) to get the re-encryption key 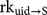 and computes 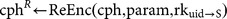.
  2. Otherwise, if there exists 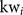 in 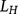 such that 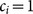 and 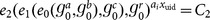, it selects 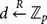, sets 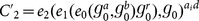, 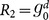 and 
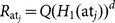
 for each 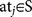, and returns 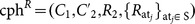;
  3. Otherwise, it reports failure and terminates.

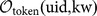
: Given a user identity 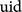 and a keyword 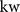, the challenger proceeds as follows:
  1. It queries 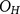 with 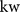 to obtain 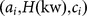.
  2. If 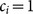, set 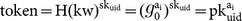;
  3. If 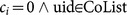, set 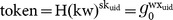;
  4. Otherwise, report failure and terminate.

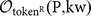
: Given an access control policy 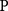 and a keyword 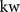, the challenger proceeds as follows:
  1. If 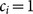, select 
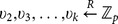
, implicitly set 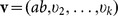 and 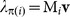 for 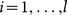. Compute for 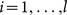,

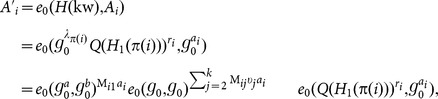


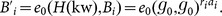

  - If 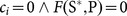, make a query 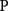 on 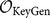 to get 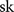, and compute 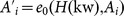 and 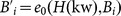 for 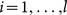.
  - Otherwise, report failure and terminate.

#### Challenge


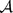
 selects an uncorrupted user 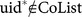 and two keywords (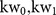) of equal length. Given 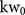 and 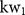, if 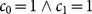, the challenger reports failure and terminates; otherwise, let 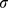 be a bit which is selected as follows:

- If 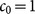 and 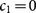, then set 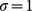,
- If 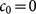 and 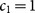, then set 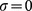,
- Otherwise, let 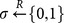.

The challenger responses 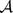 with 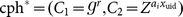.

#### Phase 2


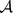
 executes the same as Phase 1.

#### Guess


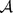
 outputs a guess 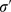. The challenger outputs 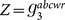 if 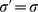; Otherwise, it outputs 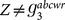.

This completes the simulation. In what follows let us analyze the probability that the challenger will not report failure and terminate due to the following two independent events:

- When 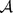 queries 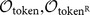 and 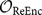, it happens that 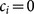 for some keyword. Note that for each query with respect to some keyword, 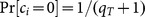. Therefore, as 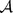 makes at most 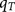 oracle queries, the probability of the challenger not reporting failure and terminating can be 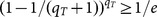.
- When 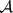 presents 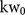 and 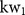, it happens that 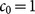 and 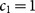. Since 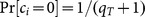 for 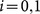, we have 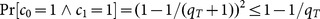. Hence, the probability that the challenger has no failure is at least 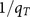.

Therefore the challenger simulates without failure with the probability at least 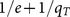.

Now let us analyze the advantage of the challenger solving 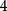-MDDH problem on condition that the simulation completes perfectly. In the challenge phase, if 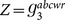, then 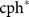 is indeed a valid ciphertext of 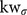. Then the probability of 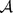 outputting 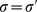 is 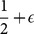. If 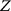 is an element randomly selected from 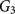, the probability of 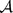 outputting 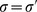 is 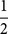. Therefore, the probability of the challenger correctly guessing 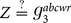 is 
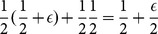
. That is, the challenger solves the 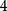-MDDH problem with advantage 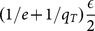 if 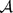 wins the selective security game with advantage 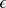. 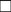


### 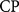 - 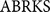 Security

Security of the 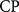 - 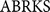 scheme can be proven as the following theorem.

#### Theorem 2

*Assume that* 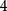 *-MDDH assumption holds, our* 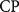 *-* 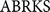 *scheme achieves selective security against chosen-keyword attack in the random oracle model.*

*Proof*: The main idea is to reduce the security of our 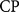-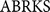 to the hardness of 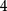-MDDH assumption. That's, we show that if there exists a probabilistic polynomial time adversary 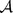 breaking the selective security game of our 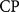-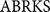 scheme against chosen-keyword attack with a non-negligible advantage 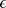, then we can construct a challenger solving 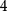-MDDH problem with a non-negligible advantage 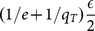, where 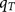 is a polynomial large number, which should be larger than the number of oracle queries for 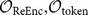 and 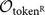. In this part, 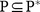 means 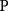 is a substructure of 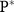.

Given an instance of 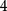-MDDH problem 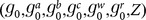 where 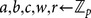 are unknown, the challenger simulates the game as follows:

#### Setup


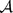
 selects a set of corrupted users denoted by 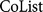 and an access control policy 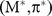, where 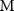 is an 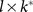 matrix, and sends them to the challenger. The challenger generates the public parameters and master key as follows:

- Given the access control policy 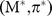, for each 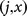 where 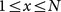 and 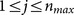, choose 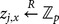. If there exists an 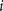 such that 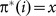 and 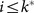, let 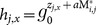; Otherwise, let 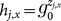.
  The public parameters is set to 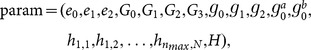
by implicitly setting the master private key 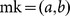.

The random oracle 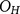 is simulated as same as the proof of Theorem 1.

#### Phase 1


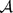
 can query the following oracles in polynomial many times:

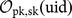
: Same as the proof of Theorem 1.

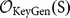
: Given an attribute set 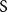, the challenger proceeds as follows:
  1. If 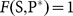, then abort.
  2. Otherwise, because 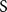 does not satisfy the access structure 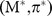, there exists a vector 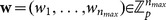 such that 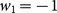 and 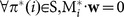. Note that we simply let 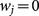 and 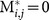 for 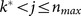. Compute 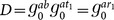 and 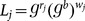 for 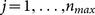, by choosing 
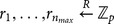
 and implicitly defining 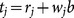, and set 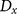 for each 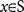 as follows:
    1. If there exists 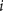 such that 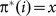, set

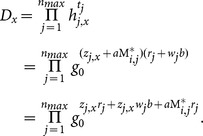

- Otherwise set 
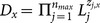
.

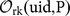
: Given a user identity 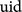 and an access control policy 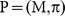, where 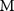 is an 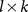 matrix, the challenger proceeds as follows:
  1. If 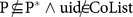, then abort.
  2. If 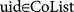, choose random elements 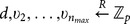, let 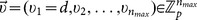, and set
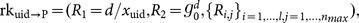
where 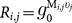.

  - Otherwise, we consider 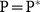 first. Choose random elements 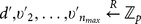 and set
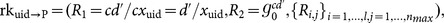
where
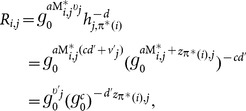
by implicitly defining 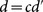 and 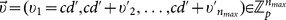
(We set 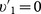). Note that the form of our re-encryption key is similar to that of the ciphertext of Water's 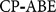 [17]. So if 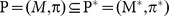, the re-encryption key can be derived from 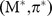 through the technology of ciphertext delegation proposed in [30].

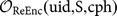
: Given a user identity 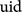, an original encrypted keyword 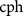 and an access control policy 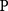, the challenger proceeds as follows:
  1. If 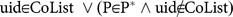, it queries 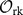 with (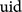, 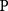) to get the re-encryption key 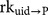 and computes 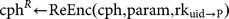.
  2. Otherwise, if there exists 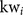 in 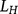 such that 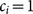 and 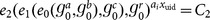, it picks 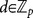, sets 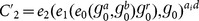, 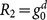 and 
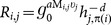
 for each 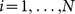 and 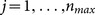, and returns 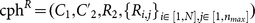;
  3. Otherwise, it reports failure and terminates.

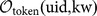
: Same as the proof of Theorem 1.

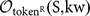
: Given an attribute set 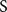 and a keyword 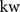, the challenger proceeds as follows:
  1. If 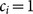, select 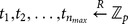. Compute

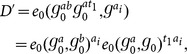
for 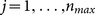, 
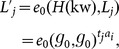
and for each 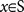, 
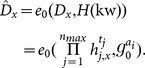

  - If 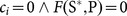, make a query 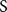 on 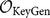 to get 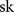, and compute 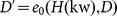, 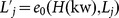 for 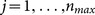 and 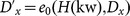 for 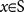.
  - Otherwise, report failure and terminate.

#### Challenge


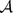
 selects an uncorrupted user 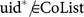 and two equal-length keywords (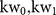). If 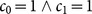, the challenger reports failure and terminates; otherwise, let 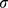 be a bit which is selected as follows:

- If 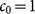 and 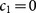, then set 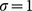,
- If 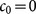 and 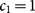, then set 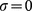,
- Otherwise, let 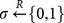.

The challenger responses 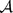 with 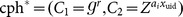.

#### Phase 2


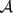
 executes the same as Phase 1.

#### Guess


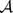
 outputs a guess 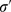. The challenger outputs 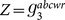 if 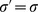. Otherwise, it outputs 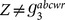.

This completes the simulation. We can show that the challenger solves the 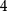-MDDH problem with advantage 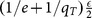 if 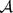 wins the selective security game of 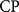-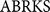 with advantage 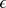 similar to the analysis of Theorem 1. 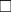


## Application

**Figure 3:**
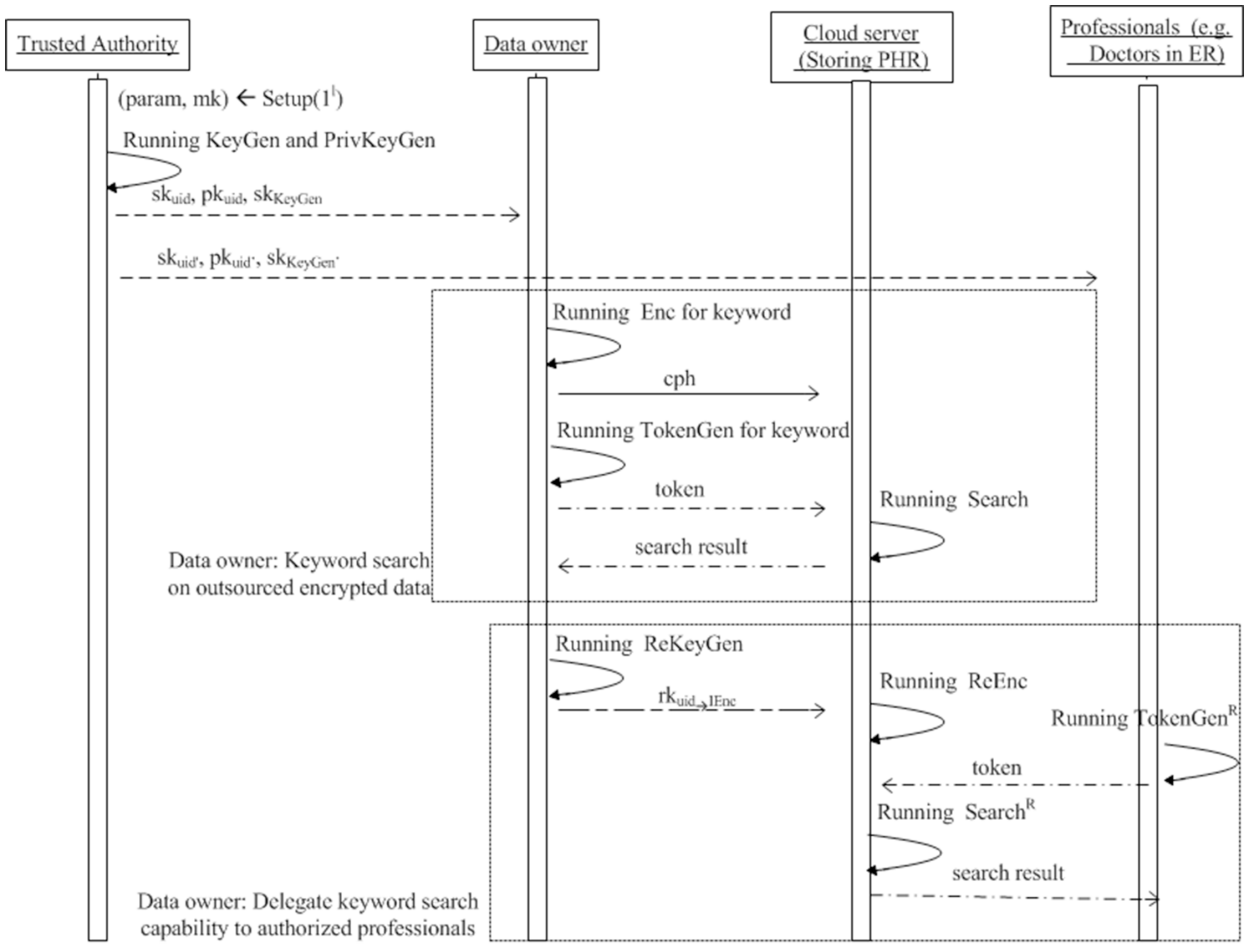
Sequence diagram for using 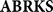 in the application where the data owner shares his medical records with some professionals such that only authorized professionals can retrieve medical records of their interests.

Our 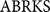 schemes fit very well for many applications in the cloud computing environment. One of the prominent applications is about Personal Health Records (PHR) for patients: The data owner encrypted his own health records and outsourced these encrypted records to the cloud which hosts the PHR service. The data owner always needs to fetch the related health records upon some keywords since it is too costly to download all encrypted records and decrypt them to get desired records. In addition, the data owner might need to share these encrypted health records with some professionals, for example, heart doctors in Emergency Room. In order to attain this goal, the data owner has to delegate the search capability. Fig. 3 shows the sequence diagram that how the entities in the PHR application make use of the proposed 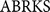 schemes to achieve these goals.

## Conclusions

In this paper, we propose a novel notion called attribute-based proxy re-encryption with keyword search (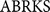). Our solutions can be used in the cloud setting, such that (1) a data owner can delegate the search capability to a group of users by specifying fine-grained access control policies; (2) the data owner and data users can delegate the tedious re-encryption and search process to the cloud without compromising data confidentiality.
